# Functional reassessment of extended splice region variants in *MYO7A* with hearing loss and Usher syndrome

**DOI:** 10.1002/path.70048

**Published:** 2026-03-19

**Authors:** Tao Shi, Yu Huang, Xiaohuan Su, Lisheng Yu, Yixin Zhao, Jing Cheng

**Affiliations:** ^1^ Department of Otorhinolaryngology – Head and Neck Surgery Peking University People's Hospital Beijing PR China; ^2^ Department of Otorhinolaryngology, West China Hospital, West China Medical School Sichuan University Chengdu PR China; ^3^ Institute of Rare Diseases, West China Hospital of Sichuan University Chengdu PR China; ^4^ Genomics Center, Core Facility of West China Hospital Sichuan University Chengdu PR China

**Keywords:** *MYO7A*, splice region, minigene assay, variant interpretation, hearing loss, Usher syndrome

## Abstract

*MYO7A* is a causal gene, underlying Usher syndrome type 1B (USH1B) and both autosomal recessive (DFNB2) and dominant (DFNA11) non‐syndromic hearing loss. Despite the large number of reported *MYO7A* variants (over 2,200), variants located in an extended splice region remain difficult to interpret and are often classified as variants of uncertain significance (VUS). We investigated the clinical impact of *MYO7A* extended splice region variants, located within ±50 bp of exon–intron boundaries, by analyzing a nationwide Chinese cohort of 10,664 undiagnosed individuals with hearing loss. Twelve such variants (in 11 probands, two variants were in *cis*) were identified for functional analysis. Using minigene splicing assays coupled with *in silico* splicing predictions, we evaluated each variant's effect on pre‐mRNA processing and applied ACMG/AMP guidelines for classification. Six of the tested variants completely disrupted normal splicing, and eight variants in total were reclassified from VUS to pathogenic or likely pathogenic based on aberrant transcript outcomes. Notably, several variants generated multiple distinct abnormal transcripts, and two‐thirds of these variants fell within the myosin motor domain (others in the FERM2 domain). Splicing predictions from *in silico* algorithms were largely concordant with the experimental results, further supporting their utility in variant interpretation. This functional evidence enabled definitive molecular diagnoses in previously unresolved cases spanning DFNA11, DFNB2, and USH1B phenotypes. In summary, our study demonstrated that integrating experimental splicing assays with predictive tools can definitively determine the pathogenicity of extended splice region variants in *MYO7A*, thereby improving the diagnostic accuracy of genetic testing for both non‐syndromic and syndromic hearing loss. This approach could be applied to other genes to enhance genetic diagnosis. © 2026 The Author(s). *The Journal of Pathology* published by John Wiley & Sons Ltd on behalf of The Pathological Society of Great Britain and Ireland.

## Introduction

Mutations in *MYO7A*, located on chromosome 11q13.5, cause a spectrum of hereditary hearing‐loss disorders, including syndromic Usher syndrome type 1B (USH1B) and non‐syndromic forms DFNB2 and DFNA11 [1, 2, 3, 4, 5]. The *MYO7A* gene encodes myosin VIIa, an unconventional motor protein expressed in the cochlea, vestibular system, and retina, where it plays a central role in stereocilia mechanotransduction and photoreceptor integration [6, 7, 8]. Defects in myosin VIIa disrupt auditory, vestibular, and visual function, giving rise to phenotypes ranging from congenital severe‐to‐profound deafness with retinitis pigmentosa (USH1B) to isolated progressive hearing loss (DFNA11, DFNB2) [9].

Accurate pre‐mRNA splicing depends on multiple *cis*‐regulatory elements, including the 5’ donor and 3’ acceptor splice sites, exonic splicing enhancers and silencers, the polypyrimidine tract, and the branch point. Single‐nucleotide variants and small insertions/deletions in or near these elements can disrupt exon definition, activate cryptic splice sites, or weaken authentic splice sites, often leading to aberrant transcripts and disease. While variants affecting the essential ±1,2 dinucleotides are generally expected to impair splicing, variants outside these positions are harder to interpret because splicing regulation is context‐dependent and incompletely captured by current prediction models [10, 11, 12, 13, 14, 15].

More than 2,200 *MYO7A* variants have been reported, including over 160 that affect splicing. Variants at ±1,2 positions are usually classified as pathogenic (P) or likely pathogenic (LP) due to predictable loss of normal transcripts [16, 17, 18]. Here, extended splice region variants are defined as variants located within ±50 bp of exon–intron boundaries, excluding the canonical ±1,2 splice sites. In contrast, variants located in extended splice regions are substantially more challenging to interpret [12]. Although numerous *MYO7A* extended splice region variants have been catalogued in ClinVar and the Leiden Open Variation Database (LOVD), the majority remain classified as variants of uncertain significance (VUS). This diagnostic ambiguity hampers genetic counseling and limits clinical utility.

Functional RNA assays can directly address this gap. In particular, minigene splicing assays provide experimentally grounded evidence of splice disruption and are recognized within the American College of Medical Genetics and Genomics and the Association for Molecular Pathology (ACMG/AMP) and ClinGen SVI frameworks for variant interpretation [19]. Numerous studies have now deployed the minigene assay to evaluate splice‐variant function, substantially reducing diagnostic uncertainty and refining patient eligibility for gene‐targeted therapies [20, 21, 22, 23]. However, cohort‐scale functional reassessment of *MYO7A* extended splice region variants remains limited.

Here, we evaluate *MYO7A* extended splice region variants within ±50 bp of exon–intron boundaries identified through the Chinese Deafness Genetics Consortium (CDGC), which includes 22,125 individuals with hearing loss. (Note: the CDGC is a national cooperative network of medical genetics research on hearing impairment and related disorders in PR China. The nomenclature has been validated; the database is publicly accessible at https://gdcdb.net/). Our analyses focused on the 10,664 genetically unsolved individuals who underwent whole‐genome sequencing. We combined minigene assays with *in silico* splicing predictions to reclassify extended splice region variants and assess their clinical relevance. By generating functional evidence for variants that would otherwise remain VUS, this study aims to improve molecular diagnosis across *MYO7A*‐related phenotypes and to provide a practical framework for interpreting extended splice region variants in hearing‐loss genes more broadly.

## Materials and methods

### Ethics approval and patient consent

This study was approved by the Ethics Committee of West China Hospital (No. 2020‐606). Written informed consent was obtained from all participants or their legal guardians, including parental consent for minors.

### Study cohort

The Chinese Deafness Genetics Consortium (CDGC), established in 2013, investigates the genetic basis of hearing loss and related syndromes. Participants were recruited from special education schools, pediatric rehabilitation centers, and hospitals across all 31 provincial‐level divisions in mainland PR China. In this study, *MYO7A* variants were identified through whole‐genome sequencing (WGS) from 10,664 undiagnosed individuals with hearing loss [pure tone audiometry (PTA) > 40 dB] who remained undiagnosed after screening of 201 known hearing loss genes. WGS was performed at the Genomic Center of West China Hospital Research Core Facility using the MGI DNBSEQT7 sequencer (BGI, Shenzhen, Guangdong, PR China).

Clinical information and peripheral blood samples were collected from probands and their family members. Diagnosis followed the clinical criteria for USH1B, including severe‐to‐profound bilateral sensorineural hearing loss, absence of vestibular responses, and presence of retinitis pigmentosa [9].

### Variant selection and filtering

**Figure 1:**
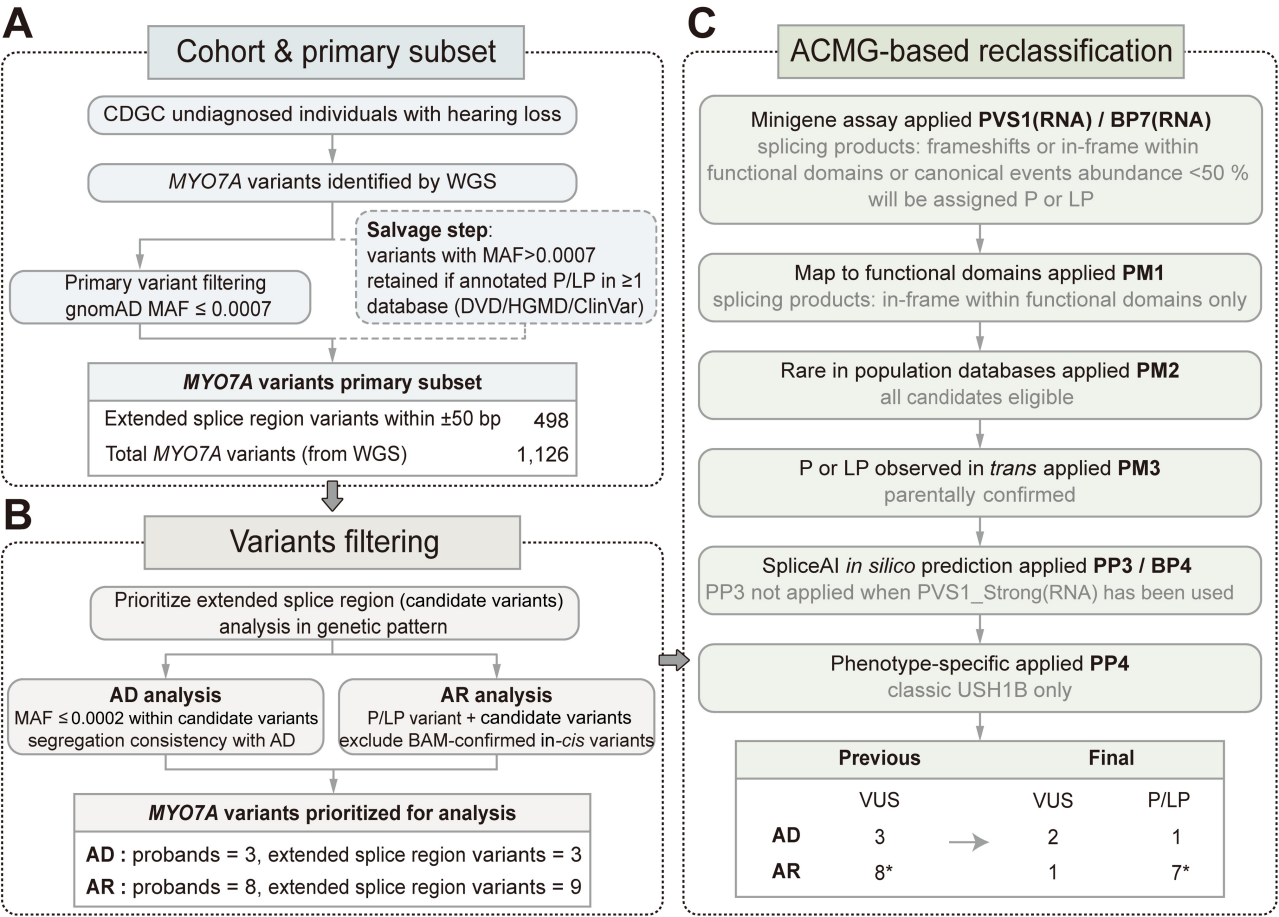
Strategy for selecting *MYO7A* extended splice region variants in the CDGC cohort. (A) Schematic representation of the strategy used for selecting *MYO7A* extended splice region variants from the Chinese Deafness Genetics Consortium (CDGC). Variants with a minor allele frequency (MAF) ≤ 0.07% and classified as likely pathogenic (LP) or pathogenic (P) in at least one database were included. (B) The distribution of *MYO7A* extended splice region variants in the study population, including the clinical phenotypes of selected probands. Probands with autosomal recessive (AR) or autosomal dominant (AD) inheritance patterns were then selected. (C) Splice region variants were included in the analytic set, followed by minigene assays for splicing analysis and ACMG‐based variant reclassification. DVD, Deafness Variation Database; HGMD, Human Gene Mutation Database; DP, depth of coverage; GQ, genotype quality; IVS, intervening sequence. *Two variants were in *cis*, and they were jointly analyzed for pathogenicity.

*MYO7A* variants were identified by WGS. We first retained rare variants with minor allele frequency (MAF) ≤ 0.0007. To maximize sensitivity, we performed a database‐guided salvage step by re‐including variants with MAF > 0.0007 if annotated as P or LP in the Deafness Variation Database (DVD) (https://deafnessvariationdatabase.org/, last accessed 30 January 2025), the Human Gene Mutation Database (HGMD) (https://www.hgmd.cf.ac.uk/ac/index.php, last accessed 30 January 2025), or ClinVar (https://www.ncbi.nlm.nih.gov/clinvar/, last accessed 30 January 2025). We then prioritized extended splice region variants within ±50 bp of exon–intron boundaries (excluding ±1,2 positions) (Figure 1A). All candidates from both the rare‐variant set and the salvage step were re‐assessed *de novo* according to ACMG/AMP and ClinGen SVI recommendations [16, 17, 18, 19]; in the absence of functional evidence, most extended splice region variants were classified as VUS at baseline [16, 17, 18, 19].

### Minigene assay

To evaluate the functional impact of *MYO7A* extended splice region variants, we constructed minigene vectors containing exonic and adjacent intronic regions of the *MYO7A* gene. Specific primers for each variant were designed using NCBI Primer‐BLAST, and PCR amplification was performed using gDNA from both probands and controls as templates. The PCR products were purified; digested with restriction enzymes – Xho I (Takara, Kusatsu, Shiga, Japan; 1635), BamH I (Takara, 1605), EcoR V (Takara, 1612), Nde I (Takara, 1621), Nhe I (Takara, 1622); and cloned into the pSPL3 plasmid. The recombinant plasmids were then transformed into *E. coli* DH5α competent cells, selected for positive clones, and sequenced for verification (supplementary material, Table S1).

For functional analysis, HEK293T cells (ATCC, Manassas, VA, USA; CRL‐11268) were transfected with the recombinant minigene plasmids using Lipofectamine™ 3000 Reagent (Invitrogen, Carlsbad, CA, USA; L3000015). After 48 h of transfection, total RNA was extracted using the RNA Extraction Kit (Tiangen, Beijing, PR China; DP451). cDNA synthesis was performed using the cDNA Synthesis Kit (Takara, RR047A), followed by PCR amplification with exon‐specific primers (supplementary material, Table S1). PCR products were analyzed by 2% agarose gel electrophoresis; band intensities of grayscale value were quantified using Bio‐Rad Image Lab software (Version 6.1; Bio‐Rad, Hercules, CA, USA). Products of comparable size were TA‐cloned (Takara, 3270) and Sanger‐sequenced (Sangon Biotech, Shanghai, PR China) to estimate relative abundance. Splicing patterns were assessed by the same sequencing workflow, and splice variants were identified by aligning reference and mutant sequences in Geneious (Version 4.8.4; Biomatters Ltd, Auckland, New Zealand). For concise and efficient annotation of the splicing events, we adopted the nomenclature of Lopez‐Perolio *et al* (supplementary material, Table S2) [24].

### Bioinformatics analysis

We used several bioinformatics tools to predict the impact of extended splice region variants on RNA splicing: SpliceAI (https://github.com/Illumina/SpliceAI) [25, 26], Pangolin (https://github.com/tkzeng/Pangolin) [27], MaxEntScan (https://www.genes.mit.edu/burgelab/maxent/Xmaxentscan_scoreseq.html) [28], and dbscSNV (https://sites.google.com/site/jpopgen/dbNSFP) [29].

To predict splice alterations, we utilized ENIGMA thresholds (https://enigmaconsortium.org) to assess the MaxEntScan scores and identify potential splice site disruptions [28]. Specifically, the diff score (Ref score − Alt score) was calculated, and variants with an Alt score below 6.2 were considered likely to affect splicing.

### Variant classification

The ACMG/AMP guidelines for variant interpretation were applied to reclassify *MYO7A* extended splice region variants based on the experimental minigene assay results. Variants were classified as follows: P or LP: pathogenic or likely pathogenic variants identified by functional evidence of splicing disruption; or VUS: variants of uncertain significance, which did not show conclusive splicing alterations. Based on the 2015 ACMG/AMP standards and the 2023 ClinGen SVI splicing refinement, we reclassified all extended splice region variants [16, 17, 18, 19]. The PVS1 (RNA) and BP7_Strong (RNA) criteria were applied to variants identified through the minigene assay, with the assignment of pathogenicity grade strictly controlled. The PVS1 (RNA) and BP7_Strong (RNA) criteria from the ACMG/AMP guidelines were applied, with variants showing in‐frame deletions or frameshifts being classified as P or LP if they affected critical domains, such as the FERM2 or myosin motor domains. If the canonical splicing product was detectable but represented < 50% of total transcript signal by densitometry, the assay was considered to support a P or LP classification. The PP3 criterion was awarded to extended splice region variants with a default SpliceAI score greater than 0.2; this evidence was presented but not applied when minigene data already supported PVS1_Strong (RNA) or higher. Extended splice regions that map to functional domains and result exclusively in non‐truncating splicing defects were assigned the PM1 criterion. The PP4 criterion was applied exclusively when the patient's phenotype was definitively consistent with USH1.

## Results

### Selection and prioritization of *MYO7A* extended splice region variants

We analyzed *MYO7A* variation identified by WGS in the nationwide CDGC cohort. In total, 1,126 *MYO7A* variants were detected and subjected to stepwise filtering. Variants were first screened by sequencing quality and allele frequency. Rare variants with MAF ≤ 0.0007 constituted the primary candidate set. To maximize sensitivity and avoid missing clinically recognized alleles, we additionally applied a database‐guided salvage step: variants with MAF > 0.0007 were retained if annotated as P or LP in at least one public database (ClinVar, DVD, or HGMD). From the combined candidate pool, we prioritized extended splice region variants located within ±50 bp of exon–intron boundaries, excluding ±1,2 positions. This yielded 498 variants for downstream assessment (Figure 1A).

We next stratified candidates by presumed inheritance and clinical presentation (Figure 1B). For the autosomal dominant (AD) track, we initially identified AD‐compatible extended splice region variants meeting stringent rarity criteria (MAF ≤ 0.0002); after applying phenotype consistency and prioritization rules, three probands carrying three extended splice region variants were selected. For the autosomal recessive (AR) track, we prioritized probands carrying two or more *MYO7A* variants, including at least one extended splice region with a second variant previously classified as P or LP. This yielded eight probands for focused functional evaluation.

**Figure 2:**
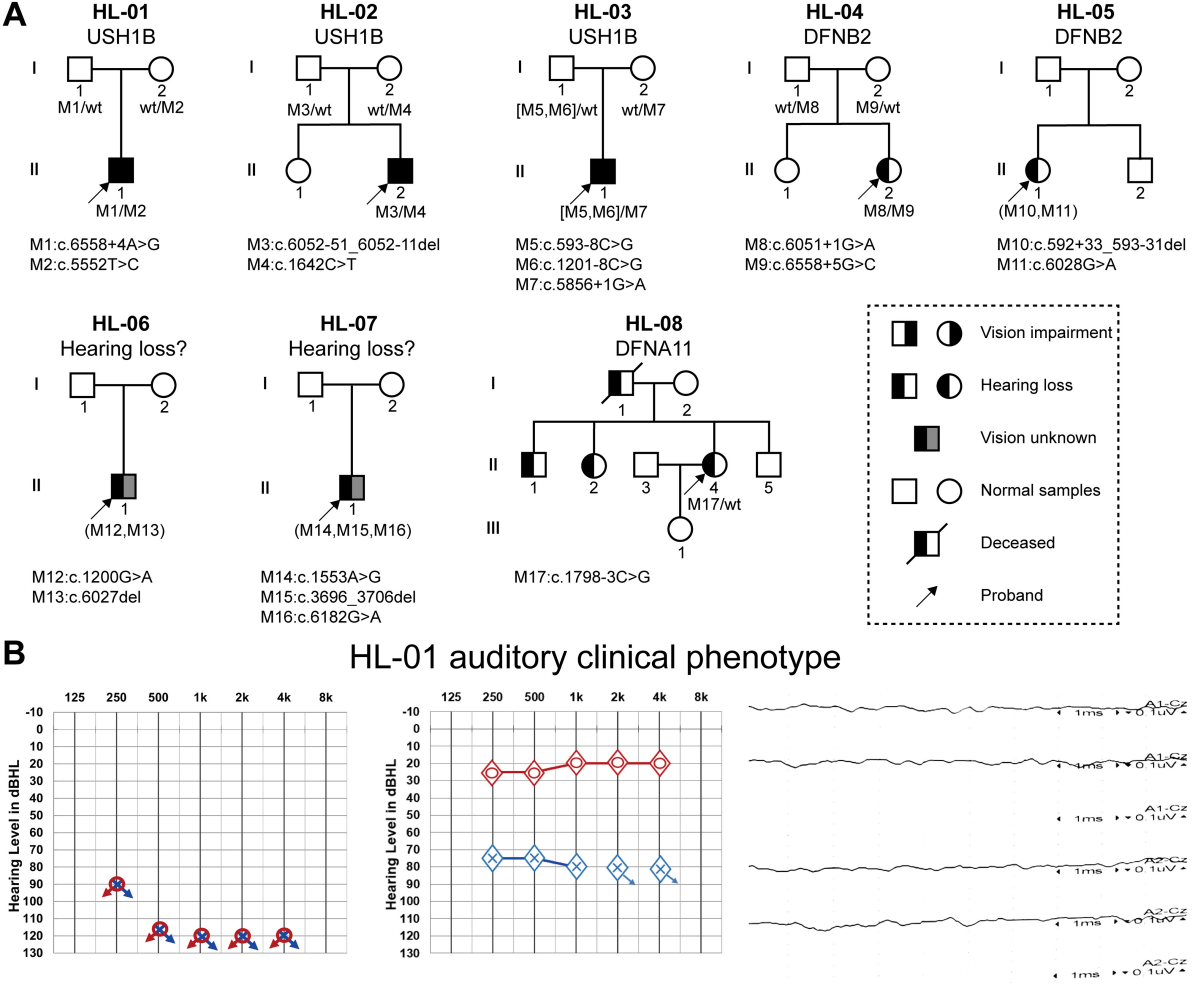
Clinical and genetic data of eight *MYO7A*‐related families. (A) Pedigree of *MYO7A*‐related patients. The clinical diagnosis is shown at the top, while the genotype of the proband is at the bottom. ‘Hearing loss?’ indicates probands for whom additional clinical information was unavailable due to loss to follow‐up. (B) Audiograms for USH1B patient HL‐01 show profound bilateral hearing loss and successful cochlear implantation (CI) in the right ear. The right panel corresponds to absent click‐ABR waves, indicating no response at 90 dB normal hearing level (nHL). A1‐Cz represents the response from the left ear, and A2‐Cz represents the response from the right ear, both measured with respect to the central electrode (Cz) on the scalp. The *x*‐axis represents time in milliseconds (ms). The *y*‐axis represents amplitude in microvolts (μV).

In total, 11 probands carrying 20 *MYO7A* variants, including 12 extended splice region variants (two of which were confirmed to be in *cis* by parental testing), were included in the analytic set (Figure 1C). Eight probands are highlighted in the main text and Figure 2 because their extended splice region variants were functionally supported for upgrade to P or LP; the remaining three probands, whose variants lacked sufficient evidence for upgrade, are presented in supplementary material, Figure S1.

### Clinical characteristics of variant carriers

Among the 11 enrolled probands, eight were reclassified as carrying P or LP extended splice region variants based on integrated functional and computational evidence. Seven of these eight probands were presumed to have an AR inheritance pattern, while one was presumed to have an AD inheritance pattern (Table 1 and Figure 2A). These eight families comprised three patients with USH1, two patients with DFNB2, and one patient with DFNA11; two additional probands had limited clinical data due to incomplete follow‐up. The DFNA11 patient exhibited late‐onset, progressive hearing loss starting at age 20 years. The two DFNB2 patients presented with congenital, profound hearing loss. All three USH1B patients showed congenital severe‐to‐profound hearing loss with bilateral myopia; two reported night blindness during their first decade, and two were diagnosed with retinitis pigmentosa (RP) during childhood. The parents of all USH1B patients reported poor balance in terms of development, and there was a significant delay in the onset of walking at 20 and 24 months of two USH1B patients, respectively (Figure 2B). The other three probands carried extended splice region variants that remained VUS after functional evaluation. One was presumed to have an AR inheritance pattern, while the other two were presumed to have an AD inheritance pattern (Table 1 and supplementary material, Figure S1A,B).

**Table 1:** Clinical and genetic summary of probands with *MYO7A* extended splice region variants.

ID	Clinical diagnosis	HL severity	Auditory onset age	Visual symptom	Visual onset age	Vestibular symptom	Variant 1 (cDNA/protein)^†^	Variant 2 (if any)^†^	Co‐segregation
HL‐01	USH1B	Profound/profound	Congenital	RP	10 years	DD	c.6558+4A>G	c.5552T>C, p.Leu1851Pro	Yes
HL‐02	USH1B	Severe/severe	Congenital	Myopia	Unknown	Equilibrium anomaly	c.6052‐51_6052‐11del	c.1642C>T, p.Gln548*	Yes
HL‐03	USH1B	Profound/profound	Congenital	RP, myopia	Childhood	DD	[c.593‐8C>G, c.1201‐8C>G]	c.5856+1G>A	Yes
HL‐04	DFNB2	Profound/profound	Congenital	Normal	/	Normal	c.6558+5G>C	c.6051+1G>A	Yes
HL‐05	DFNB2	Profound/profound	Congenital	Normal	/	Normal	c.592+33_593‐31del	c.6028G>A, p.Asp2010Asn	NA
HL‐06	HL?^‡^	Profound/profound	NA	NA	NA	NA	c.1200G>A, p.Lys400=	c.6027del, p.Asp2010Ilefs*31	NA
HL‐07	HL?^‡^	Profound/profound	NA	NA	NA	NA	c.1553A>G, p.Lys518Arg	c.3696_3706del, p.Arg1232Serfs*72	NA
								c.6182G>A, p.Arg2061Gln	
HL‐08	DFNA11	Progressive	Post‐lingual	Normal	/	Normal	c.1798‐3C>G	/	NA
HL‐S1	HL?^‡^	NA	Congenital	NA	NA	NA	c.3375+34G>A	c.2075_2079delinsGTA, p.Val692Glyfs*18	NA
HL‐S2	HL?^‡^	Severe/severe	Post‐lingual	Normal	/	Normal	c.5043+3A>G	/	NA
HL‐S3	HL?^‡^	Moderately severe/moderately severe	Post‐lingual	Normal	/	Normal	c.3630+45C>T	/	NA

The “/” indicated that the proband did not have any visual abnormalities.

^†^ HGVS (Human Genome Variation Society) nomenclature using NM_000260.4 as a reference.

^‡^ HL? (hearing loss). Due to loss to follow‐up, the determination of other phenotypes was not possible.

DD, developmental delay; RP, retinitis pigmentosa.

### Minigene assay results

**Figure 3:**
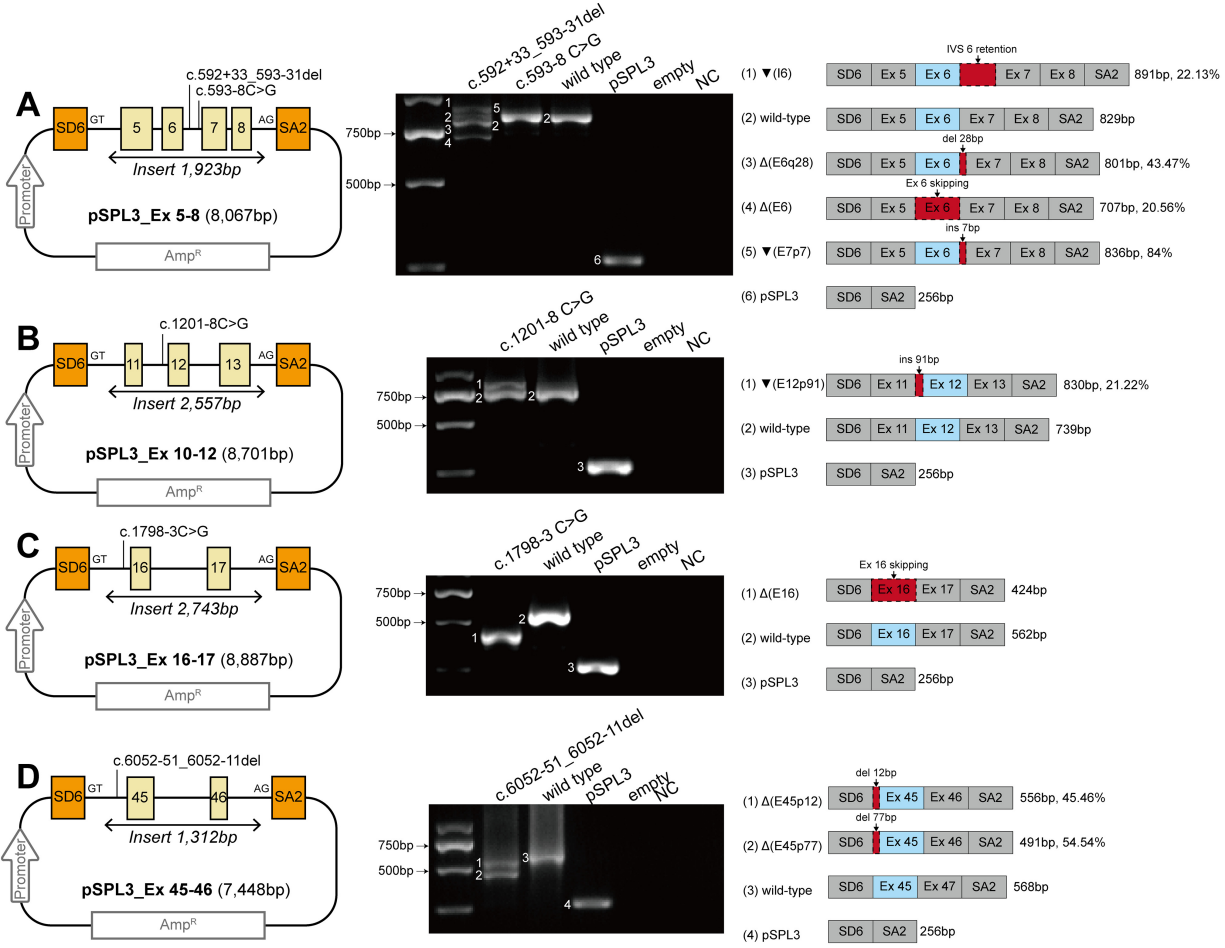
Splice defects induced by *MYO7A* acceptor site extended splice region variants in minigene assays. (A) *MYO7A* exons 5–8; (B) exons 11–13; (C) exons 16–17; (D) exons 45–46. For every assay and composite panel: Left: variant‐specific minigene construct: pSPL3 with SV40 promoter, ampicillin resistance gene (Amp^R^), pSPL3 vector‐specific exons (orange boxes), *MYO7A* exons (yellow boxes), and location of variants tested in each assay. Middle: RT‐PCR products resolved on agarose gel. From left to right: size standard; variant(s) minigene(s); wild‐type minigene; empty pSPL3; non‐transfected HEK293T; and no‐template control (NC). Numbered bands correspond to the schematic on the right. Note that not all splicing products identified can be visible, such as variant c.593‐8C>G. Right: schematic representation of observed splicing products, showing product lengths and percentage densitometry of all bands generated by the same variants.

**Figure 4:**
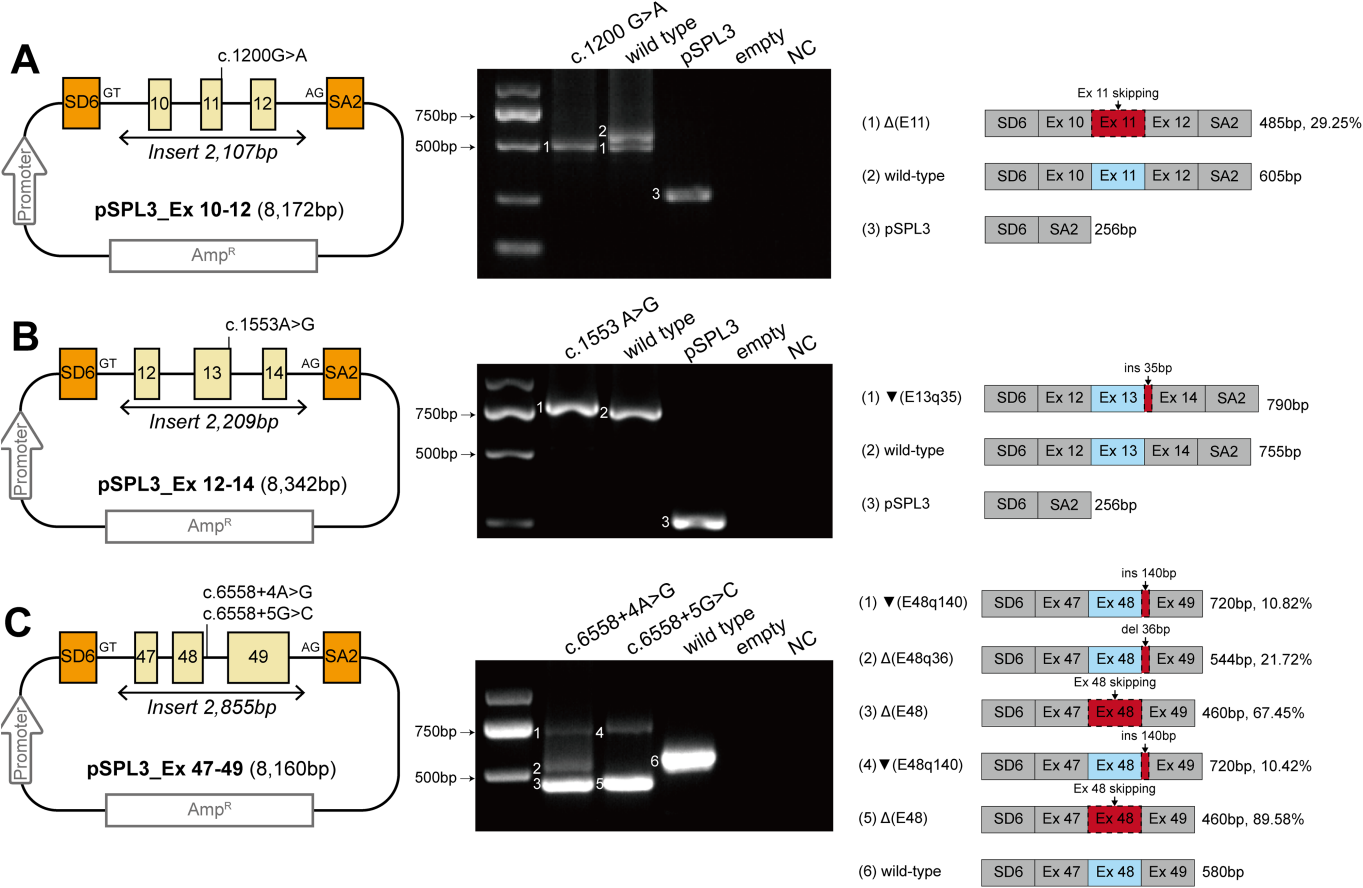
Splice defects induced by *MYO7A* donor site extended splice region variants in minigene assays. (A) *MYO7A* exons 10–12; (B) exons 12–14; (C) exons 47–49. For every assay and composite panel: Left: variant‐specific minigene construct: pSPL3 with SV40 promoter, ampicillin resistance gene (Amp^R^), pSPL3 vector‐specific exons (orange boxes), *MYO7A* exons (yellow boxes), and location of variants tested in each assay. Middle: RT‐PCR products resolved on agarose gel. From left to right, size standard, variant(s) minigene(s), wild‐type minigene, empty pSPL3, non‐transfected HEK293T, no‐template control (NC). Numbered bands correspond to the schematic on the right. Right: schematic representation of observed splicing products with product lengths and the percentage densitometry of all bands generated by the same variants. Note that for the variants 6558+4A>G and 6558+5G>C, the primer pair spans exon 49, which is the terminal exon of *MYO7A*.

Minigene assays were performed to evaluate the splicing effects of nine prioritized *MYO7A* extended splice region variants (Figures 3 and 4; supplementary material, Table S2). All tested variants resulted in at least one aberrant transcript. Notably, the variants c.592+33_593‐31del, c.6558+4A>G, and c.6558+5G>C caused multiple distinct splicing events, including exon skipping, intron retention, and altered splice site usage. The variant c.592+33_593‐31del produced the highest frequency of splicing disruptions, affecting multiple splice sites and causing exon 6 skipping and intron 6 retention.

Of the nine extended splice region variants tested, 66.67% (6/9) were located within the myosin motor domain, and 33.33% (3/9) were located within the FERM2 domain (supplementary material, Figure S2). Three variants resulted in frameshift mutations, while five variants caused in‐frame deletions, all occurring in the myosin motor or FERM2 domains.

Two wild‐type minigene constructs showed exon 11 or exon 26 skipping (Figure 4A and supplementary material, Figure S1C and Table S2). However, RT‐PCR of mouse cochleae at E16, P0, and P7 detected only full‐length transcripts (supplementary material, Figure S3), indicating that these minigene‐derived skipping events do not occur endogenously.

### *In silico* splicing predictions

We compared the results of SpliceAI, Pangolin, MaxEntScan, and dbscSNV with the minigene assay results. For variants confirmed to cause aberrant splicing, SpliceAI and Pangolin yielded similar predictions, with high prediction scores (SpliceAI Δscore > 0.2, Pangolin score > 0.2) for all but c.1201‐8C>G (supplementary material, Table S3). Notably, when the SpliceAI evaluation window was extended to 500 bp, c.1201‐8C>G reached a score of 0.23, consistent with experimentally confirmed splicing alteration.

MaxEntScan predicted splicing disruption for the variant c.1201‐8C>G, which was consistent with the minigene assay results. However, MaxEntScan's detection range was narrower than those of SpliceAI and Pangolin, and it could not evaluate the splicing impact of small intronic deletions, such as the variants c.592+33_593‐31del and c.6052‐51_6052‐11del. For variants classified as benign or likely benign, all four tools correctly predicted a lack of significant splicing impact for variants with MAF > 0.03% (supplementary material, Table S4).

### ACMG/AMP reclassification

Before functional testing, all extended splice region variants were classified as VUS based on computational evidence (e.g. SpliceAI‐supporting PP3). After minigene assays, we incorporated RNA‐based criteria for reclassification, applying PVS1 (RNA) to variants demonstrating splice disruption with predicted deleterious consequences and BP7_Strong (RNA) to variants showing no meaningful splicing impact. Variants that produced aberrant transcripts resulting in frameshifts or in‐frame deletions affecting critical functional domains were upgraded to P or LP. When canonical splicing products were still detectable, we used band densitometry to estimate residual normal transcripts; variants with less than 50% canonical product were assigned P/LP‐level functional evidence. For variants located in established functional domains that caused non‐truncating in‐frame splicing alterations, we additionally considered PM1.

**Figure 5:**
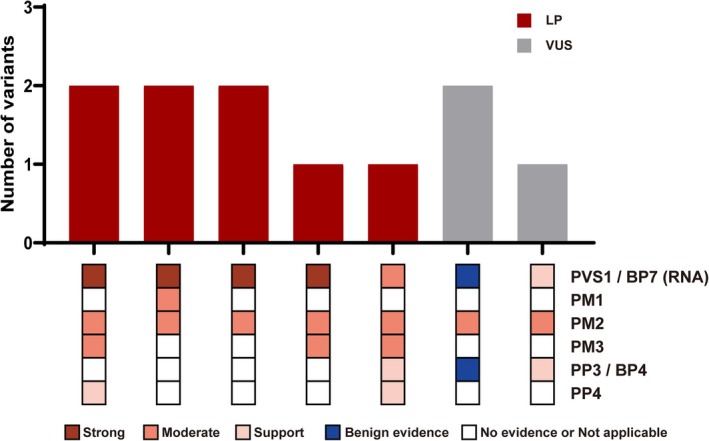
Summary of the ACMG classification for *MYO7A* extended splice region variants. The distribution of six distinct ACMG evidence codes across variant classifications. Stacked bars indicate the number of variants, and the six small squares beneath each bar correspond to the five ACMG evidence categories. These two variants were in *cis* and were therefore analyzed together for pathogenicity, resulting in an ACMG reclassification of LP.

Using this integrated framework, eight variants were reclassified as P or LP, while three remained VUS (Table 2, Figure 5, and supplementary material, Table S5). In general, variants meeting PM2 and supported by minigene evidence at or above a PVS1_Moderate (RNA) level were consistently classified as likely pathogenic (Figure 5). Although c.5043+3A>G generated two frameshift transcripts, its splicing pattern did not fully align with the canonical pathogenic mechanism proposed for DFNA11. Therefore, we conservatively applied PVS1_Supporting (RNA).

**Table 2:** ACMG/AMP classification of *MYO7A* extended splice region variants.

c.HGVS*	ACMG/AMP^†^	PVS1 (RNA)/BP7 (RNA)^‡^	PM1^§^	PM2	PM3	PP3/BP4^||^	PP4	ClinVar^¶^
c.6558+4A>G	LP	PVS1_Strong (RNA)	NA	+	+	PP3	+	Not reported
c.6052‐51_6052‐11del	LP	PVS1_Strong (RNA)	NA	+	+	PP3	+	Not reported
[c.593‐8C>G, c.1201‐8C>G]**	LP	PVS1_Moderate (RNA)	NA	+	+	PP3	+	Not reported
c.6558+5G>C	LP	PVS1_Strong (RNA)	NA	+	+	PP3	+	Not reported
c.592+33_593‐31del	LP	PVS1_Strong (RNA)	NA	+	NA	PP3	+	Not reported
c.1200G>A	LP	PVS1_Strong (RNA)	+	+	NA	PP3	NA	Not reported
c.1553A>G	LP	PVS1_Strong (RNA)	NA	+	NA	PP3	NA	Not reported
c.1798‐3C>G	LP	PVS1_Strong (RNA)	+	+	NA	PP3	+	VUS
c.3375+34G>A	VUS	BP7_Strong (RNA)	NA	+	NA	BP4	NA	Not reported
c.5043+3A>G	VUS	PVS1_Support (RNA)	NA	+	NA	PP3	+	Not reported
c.3630+45C>T	VUS	BP7_Strong (RNA)	NA	+	NA	BP4	NA	Not reported

This table presents only the ACMG/AMP evidence that contributed to the final classification. For further details, see supplementary material, Tables S2–S5.

* HGVS (Human Genome Variation Society) nomenclature using NM_000260.4 as a reference.

^†^ P, pathogenic; LP, likely pathogenic; VUS, variant of uncertain significance.

^‡^ PVS1 (RNA) and BP7 (RNA) code strength derived from minigene assay. For further details, see supplementary material, Table S2.

^§^ PM3 code restricted to variants located in key domains and causing in‐frame alterations.

^||^ We have applied PP3 and BP4 code based on the SpliceAI score (Δ score). For further details, see supplementary material, Table S3.

^¶^ For comparative purposes, we summarize the ClinVar status of the *MYO7A* extended splice region variants under investigation (last accessed 25 October 2025).

** The two variants are in *cis*.

## Discussion

In this study, we systematically evaluated extended splice region variants, which are located within ±50 bp of exon–intron boundaries, in *MYO7A* in a large Chinese hearing‐loss cohort and reclassified eight variants from VUS to P or LP by integrating minigene splicing assays with *in silico* predictions. These findings support extended splice region variants as clinically important contributors to the allelic spectrum of USH1B, DFNB2, and DFNA11, and demonstrate that functional splicing assays can substantially reduce diagnostic uncertainty for *MYO7A*‐associated disease. To our knowledge, the RNA splicing outcomes reported here have not been previously documented for these specific variants in the literature or in ClinVar, highlighting the novelty and clinical utility of the dataset.

A central strength of this work is the use of a minigene framework to generate direct experimental evidence for splice disruption among extended splice region variants outside the ±1,2 positions. Importantly, this approach preserves the local genomic context relevant to splicing – authentic splice sites, flanking intronic sequence, and adjacent exons – while providing a controlled system for comparing wild‐type and mutant constructs [30, 31]. In practice, this enables scalable functional assessment when patient‐derived RNA is unavailable, which is common for historical samples and for disorders where the most relevant tissues (e.g. inner ear and retina) are inaccessible. Nevertheless, functional readouts should be interpreted within their clinical context as part of variant‐level interpretation and, where feasible, complemented by analyses using RNA from accessible patient‐derived tissues (e.g. blood or fibroblasts) or disease‐relevant models.

We also observed exon 11 and exon 26 skipping in two wild‐type minigene constructs. Because these isoforms have been reported as naturally occurring alternative splicing events in human samples (SpliceVault database), we describe them as alternative splicing. The absence of corresponding isoforms in our mouse cochlear RT‐PCR does not exclude their presence in human cochlea or retina, and may reflect tissue‐ and species‐specific splicing regulation. This highlights the importance of interpreting minigene results alongside external transcriptomic resources and validating key isoforms in disease‐relevant human tissues when possible.

Beyond variant classification, our data are consistent with the emerging view that spliceosome ‘spatial constraints’ can be a major determinant of splice failure, particularly for intronic deletions that leave canonical dinucleotides intact. For example, c.592+33_593‐31del occurs in a short intron and may reduce the donor‐to‐branch‐point architecture below a functional threshold, whereas c.6052‐51_6052‐11del is predicted to remove core intronic elements such as the polypyrimidine tract and branch point [32]. Together, these cases illustrate how extended splice region variants can be pathogenic via disruption of intronic architecture and auxiliary regulatory elements rather than direct alteration of consensus splice dinucleotides.

We further compared multiple *in silico* tools with experimental outcomes. SpliceAI and Pangolin were generally concordant with minigene results, but c.1201‐8C>G underscores a practical limitation: under default settings, a narrow prediction window can miss cryptic splice effects. Notably, expanding the SpliceAI window (±500 bp) increased the predicted score for c.1201‐8C>G above a commonly used interpretive threshold, aligning with the experimentally confirmed splice defect. These observations support using *in silico* tools for prioritization while emphasizing that experimental validation remains critical for extended splice region variants and for borderline computational predictions.

Finally, although our sample size is limited at the level of individual phenotypic subgroups, the variants upgraded to P or LP were enriched in established functional regions of *MYO7A* (notably the myosin motor and FERM2 domains), consistent with prior genotype–phenotype observations in *MYO7A*‐associated disease [33]. Larger datasets with more complete phenotyping will be required to refine domain‐level risk estimates and to distinguish the contributions of splice consequence (frameshift versus in‐frame) from protein domain context across USH1B, DFNB2, and DFNA11.

Several limitations should be acknowledged: (1) HEK293T cells were used for minigene assays, which may not fully replicate splicing patterns in patient tissues (e.g. cochlea or retina), which have distinct splicing landscapes. (2) Patient‐derived RNA was not available for the variants studied, limiting direct *in vivo* confirmation of isoform usage and abundance. (3) Deep‐intronic pseudoexon‐creating variants were outside the scope of this work and warrant dedicated investigation in *MYO7A*.

Our results demonstrate that integrating functional splice assays with computational prediction can resolve a substantial fraction of *MYO7A* extended splice region VUS variants, improve molecular diagnosis, and provide a generalizable framework for splicing‐variant interpretation in hereditary hearing loss and related syndromes [34, 35, 36].

## Author contributions statement

All authors contributed to the study conception and design. Material preparation, data collection and analysis were performed by TS and YH. Minigene assay was performed by XS. The review and editing of the manuscript and project administration were performed by JC. The first draft of the manuscript was written by TS, and all authors commented on previous versions of the manuscript. All authors read and approved the final manuscript.

## Supporting information


**Figure S1.** Pedigrees and minigene results for patients carrying VUS extended splice region variants
**Figure S2**. Distribution of variants across *MYO7A* exons and domains
**Figure S3**. RT‐PCR analysis of cochlear tissue of mice at different time points

**Table S1.** List of primers for minigene construction and cDNA sequencing
**Table S2**. Summary of minigene assay results for *MYO7A* extended splice region variants
**Table S3**. Overview of *MYO7A* extended splice region variants and splice prediction scores
**Table S4**. Overview of reported benign *MYO7A* extended splice region variants and splice prediction scores
**Table S5**. ACMG/AMP classification of *MYO7A* extended splice region variants before and after functional validation

## Data Availability

The datasets generated in this study have been included in the article. For enquiries or requests to access the data, please contact the corresponding author. All *MYO7A* extended splice region variants identified in this study will be publicly available in SpliceVarDB.

## References

[1] Weil D , Blanchard S , Kaplan J , *et al*. Defective myosin VIIA gene responsible for Usher syndrome type 1B. Nature 1995; 374 **:** 60–61. 7870171 10.1038/374060a0

[2] Delmaghani S , El‐Amraoui A . The genetic and phenotypic landscapes of Usher syndrome: from disease mechanisms to a new classification. Hum Genet 2022; 141 **:** 709–735. 35353227 10.1007/s00439-022-02448-7 PMC9034986

[3] Liu XZ , Walsh J , Tamagawa Y , *et al*. Autosomal dominant non‐syndromic deafness caused by a mutation in the myosin VIIA gene. Nat Genet 1997; 17 **:** 268–269. 9354784 10.1038/ng1197-268

[4] Liu XZ , Walsh J , Mburu P , *et al*. Mutations in the myosin VIIA gene cause non‐syndromic recessive deafness. Nat Genet 1997; 16 **:** 188–190. 9171832 10.1038/ng0697-188

[5] Weil D , Küssel P , Blanchard S , *et al*. The autosomal recessive isolated deafness, DFNB2, and the Usher 1B syndrome are allelic defects of the myosin‐VIIA gene. Nat Genet 1997; 16 **:** 191–193. 9171833 10.1038/ng0697-191

[6] Nisenbaum E , Thielhelm TP , Nourbakhsh A , *et al*. Review of genotype–phenotype correlations in Usher syndrome. Ear Hear 2022; 43 **:** 1–8. 34039936 10.1097/AUD.0000000000001066 PMC8613301

[7] Pan L , Yan J , Wu L , *et al*. Assembling stable hair cell tip link complex via multidentate interactions between harmonin and cadherin 23. Proc Natl Acad Sci U S A 2009; 106 **:** 5575–5580. 19297620 10.1073/pnas.0901819106 PMC2667052

[8] Underhill A , Webb S , Grandi FC , *et al*. MYO7A is required for the functional integrity of the mechanoelectrical transduction complex in hair cells of the adult cochlea. Proc Natl Acad Sci U S A 2025; 122 **:** e2414707122. 39746042 10.1073/pnas.2414707122 PMC11725811

[9] Bjb K , Lentz J . Usher Syndrome Type I: GeneReviews™, 2016. [Accessed 30 March 2025]. Available from: https://www.ncbi.nlm.nih.gov/books/NBK1265/.

[10] Plaschka C , Newman AJ , Nagai K . Structural basis of nuclear pre‐mRNA splicing: lessons from yeast. Cold Spring Harb Perspect Biol 2019; 11 **:** a032391. 30765413 10.1101/cshperspect.a032391 PMC6496352

[11] Hang J , Wan R , Yan C , *et al*. Structural basis of pre‐mRNA splicing. Science 2015; 349 **:** 1191–1198. 26292705 10.1126/science.aac8159

[12] Scotti MM , Swanson MS . RNA mis‐splicing in disease. Nat Rev Genet 2016; 17 **:** 19–32. 26593421 10.1038/nrg.2015.3 PMC5993438

[13] Wang G‐S , Cooper TA . Splicing in disease: disruption of the splicing code and the decoding machinery. Nat Rev Genet 2007; 8 **:** 749–761. 17726481 10.1038/nrg2164

[14] Ward AJ , Cooper TA . The pathobiology of splicing. J Pathol 2010; 220 **:** 152–163. 19918805 10.1002/path.2649 PMC2855871

[15] Pedrotti S , Cooper TA . In brief: (mis)splicing in disease. J Pathol 2014; 233 **:** 1–3. 24615176 10.1002/path.4337 PMC4300095

[16] Richards S , Aziz N , Bale S , *et al*. Standards and guidelines for the interpretation of sequence variants: a joint consensus recommendation of the American College of Medical Genetics and Genomics and the Association for Molecular Pathology. Genet Med 2015; 17 **:** 405–424. 25741868 10.1038/gim.2015.30 PMC4544753

[17] Abou Tayoun AN , Pesaran T , DiStefano MT , *et al*. Recommendations for interpreting the loss of function PVS1 ACMG/AMP variant criterion. Hum Mutat 2018; 39 **:** 1517–1524. 30192042 10.1002/humu.23626 PMC6185798

[18] Oza AM , DiStefano MT , Hemphill SE , *et al*. Expert specification of the ACMG/AMP variant interpretation guidelines for genetic hearing loss. Hum Mutat 2018; 39 **:** 1593–1613. 30311386 10.1002/humu.23630 PMC6188673

[19] Walker LC , Mdl H , Wiggins GAR , *et al*. Using the ACMG/AMP framework to capture evidence related to predicted and observed impact on splicing: recommendations from the ClinGen SVI splicing subgroup. Am J Hum Genet 2023; 110 **:** 1046–1067. 37352859 10.1016/j.ajhg.2023.06.002 PMC10357475

[20] Rawnsley K , Weisschuh N , Kohl S , *et al*. Comprehensive functional splicing analysis of non‐canonical *CNGB3* variants using *in vitro* minigene splice assays. J Pathol 2025; 266 **:** 322–336. 40304364 10.1002/path.6431 PMC12146803

[21] Valenzuela‐Palomo A , Bueno‐Martínez E , Sanoguera‐Miralles L , *et al*. Splicing predictions, minigene analyses, and ACMG–AMP clinical classification of 42 germline *PALB2* splice‐site variants. J Pathol 2021; 256 **:** 321–334. 34846068 10.1002/path.5839 PMC9306493

[22] Fraile‐Bethencourt E , Valenzuela‐Palomo A , Díez‐Gómez B , *et al*. Mis‐splicing in breast cancer: identification of pathogenic *BRCA2* variants by systematic minigene assays. J Pathol 2019; 248 **:** 409–420. 30883759 10.1002/path.5268

[23] Bueno‐Martínez E , Sanoguera‐Miralles L , Valenzuela‐Palomo A , *et al*. Minigene‐based splicing analysis and ACMG/AMP‐based tentative classification of 56 *ATM* variants. J Pathol 2022; 258 **:** 83–101. 35716007 10.1002/path.5979 PMC9541484

[24] Lopez‐Perolio I , Leman R , Behar R , *et al*. Alternative splicing and ACMG‐AMP‐2015‐based classification of PALB2 genetic variants: an ENIGMA report. J Med Genet 2019; 56 **:** 453–460. 30890586 10.1136/jmedgenet-2018-105834 PMC6591742

[25] de Sainte Agathe J‐M , Filser M , Isidor B , *et al*. SpliceAI‐visual: a free online tool to improve SpliceAI splicing variant interpretation. Hum Genomics 2023; 17 **:** 7. 36765386 10.1186/s40246-023-00451-1 PMC9912651

[26] Jaganathan K , Kyriazopoulou Panagiotopoulou S , McRae JF , *et al*. Predicting splicing from primary sequence with deep learning. Cell 2019; 176 **:** 535–548.e24. 30661751 10.1016/j.cell.2018.12.015

[27] Zeng T , Li YI . Predicting RNA splicing from DNA sequence using Pangolin. Genome Biol 2022; 23 **:** 103. 35449021 10.1186/s13059-022-02664-4 PMC9022248

[28] Shamsani J , Kazakoff SH , Armean IM , *et al*. A plugin for the Ensembl Variant Effect Predictor that uses MaxEntScan to predict variant spliceogenicity. Bioinformatics 2019; 35 **:** 2315–2317. 30475984 10.1093/bioinformatics/bty960 PMC6596880

[29] Liu X , Li C , Mou C , *et al*. dbNSFP v4: a comprehensive database of transcript‐specific functional predictions and annotations for human nonsynonymous and splice‐site SNVs. Genome Med 2020; 12 **:** 103. 33261662 10.1186/s13073-020-00803-9 PMC7709417

[30] Bonnet C , Krieger S , Vezain M , *et al*. Screening *BRCA1* and *BRCA2* unclassified variants for splicing mutations using reverse transcription PCR on patient RNA and an *ex vivo* assay based on a splicing reporter minigene. J Med Genet 2008; 45 **:** 438–446. 18424508 10.1136/jmg.2007.056895

[31] Wai HA , Lord J , Lyon M , *et al*. Blood RNA analysis can increase clinical diagnostic rate and resolve variants of uncertain significance. Genet Med 2020; 22 **:** 1005–1014. 32123317 10.1038/s41436-020-0766-9 PMC7272326

[32] Canson DM , Llinares‐Burguet I , Fortuno C , *et al*. *TP53* minigene analysis of 161 sequence changes provides evidence for role of spatial constraint and regulatory elements on variant‐induced splicing impact. NPJ Genom Med 2025; 10 **:** 37. 40341019 10.1038/s41525-025-00498-0 PMC12062376

[33] Joo SY , Na G , Kim JA , *et al*. Clinical heterogeneity associated with *MYO7A* variants relies on affected domains. Biomedicine 2022; 10 **:** 798. 10.3390/biomedicines10040798 PMC9028242 35453549

[34] Rosso LE , Pianigiani G , Morgan A , *et al*. Unraveling the functional impact of splicing variants in inherited hearing disorders through minigene splicing assays. Biomedicine 2025; 13 **:** 2245. 10.3390/biomedicines13092245 PMC12467345 41007806

[35] Reurink J , Oostrik J , Aben M , *et al*. Minigene‐based splice assays reveal the effect of non‐canonical splice site variants in *USH2A* . Int J Mol Sci 2022; 23 **:** 13343. 36362125 10.3390/ijms232113343 PMC9654511

[36] Brownstein Z , Kamal L , Mishan‐Montefiori S , *et al*. A multicenter study reveals a novel pathogenic splice‐site founder variant in *OTOF* . Hum Genomics 2025; 19 **:** 112. 41053850 10.1186/s40246-025-00819-5 PMC12502505

