# Supplementary material for: Functional reassessment of extended splice region variants in MYO7A with hearing loss and Usher syndrome
Source: J Pathol. 2026 Mar 19;269(2):222–31. doi: 10.1002/path.70048 (PMC13140121; doi:10.1002/path.70048)
Supplement: Supplementary file 1 — Figure S1. Pedigrees and minigene results for patients carrying VUS extended splice region variants Figure S2. Distribution of variants across MYO7A exons and domains Figure S3. RT‐PCR analysis of cochlear tissue of mice at different time points [file PATH-269-222-s001.docx]

**Functional reassessment of extended splice region variants in *MYO7A* with hearing loss and Usher syndrome**

T Shi, Y Huang, X Su *et al.* *J Pathol* [https://doi.org/10.1002/path**.**70048](https://doi.org/10.1002/path.70048)

**Supplementary Figures S1–S3**

**(Supplementary Tables S1–S5 are provided in a separate Excel file)**


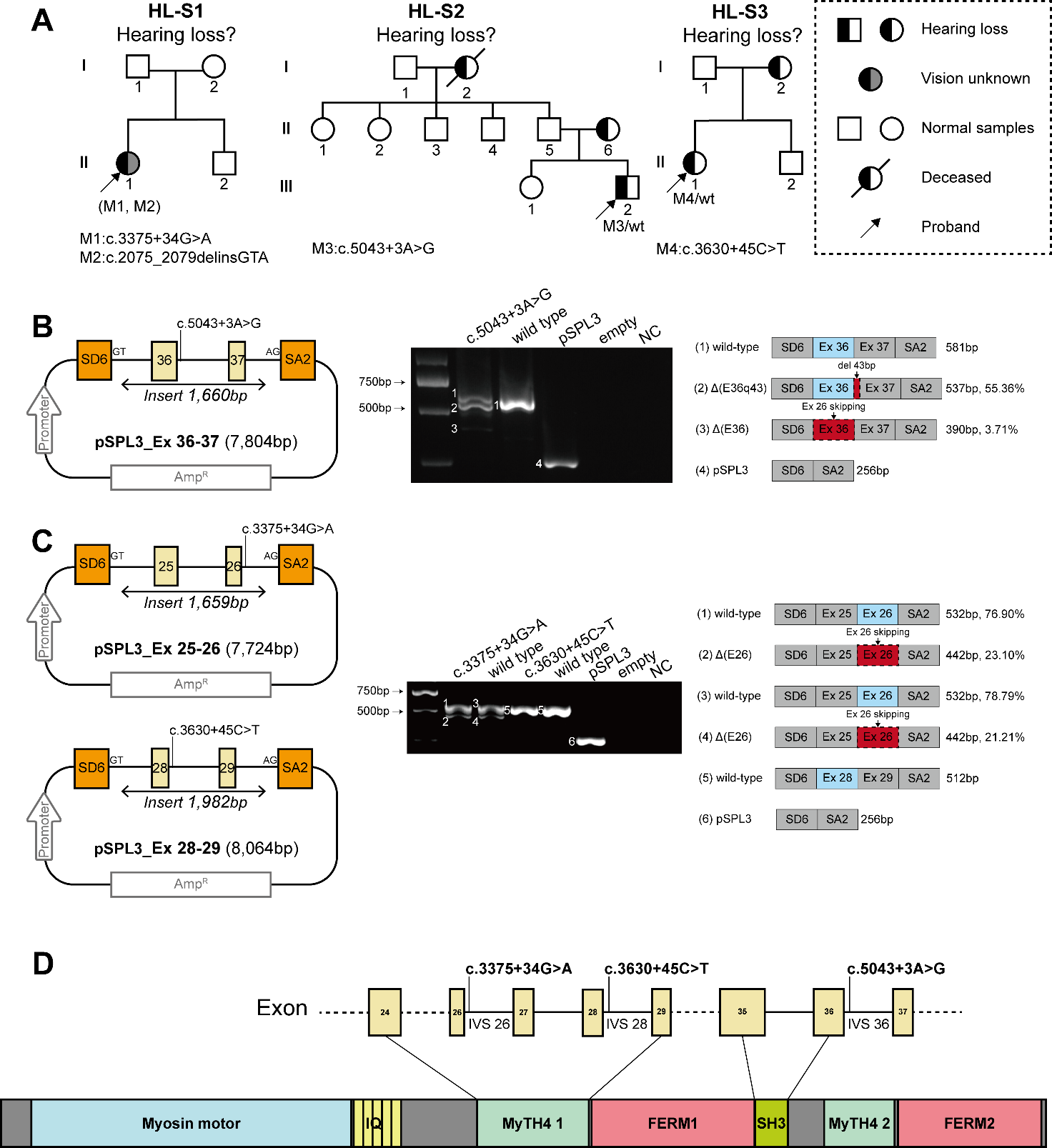


**Figure S1. Pedigrees and minigene results for patients carrying VUS extended splice region variants.** (A) Family pedigrees of *MYO7A*-related patients. At the top of each pedigree is the phenotype of the proband. ‘Hearing loss?’ indicates probands for whom additional clinical information was unavailable due to loss to follow-up. (B, C) Minigene assays in *MYO7A* exons 36–37 (B) and exons 25–26 and 28–29 (C). For each assay and composite panel: Left: variant-specific minigene construct showing pSPL3 with SV40 promoter, ampicillin resistance gene (Amp^R^), pSPL3 vector-specific exons (orange boxes), *MYO7A* exons (yellow boxes), and location of variants tested in each assay. Middle: RT-PCR products resolved on agarose gel. From left to right, size standard, variant(s) minigene(s), wild-type minigene, empty pSPL3, non-transfected HEK293T, no-template control (NC). Numbered bands correspond to the schematic on the right. Right: schematic representation of observed splicing products with product lengths and the percentage densitometry of all bands generated by the same variants. (D) Distribution of VUS or LB extended splice region variants across *MYO7A* exons and protein domains.

**
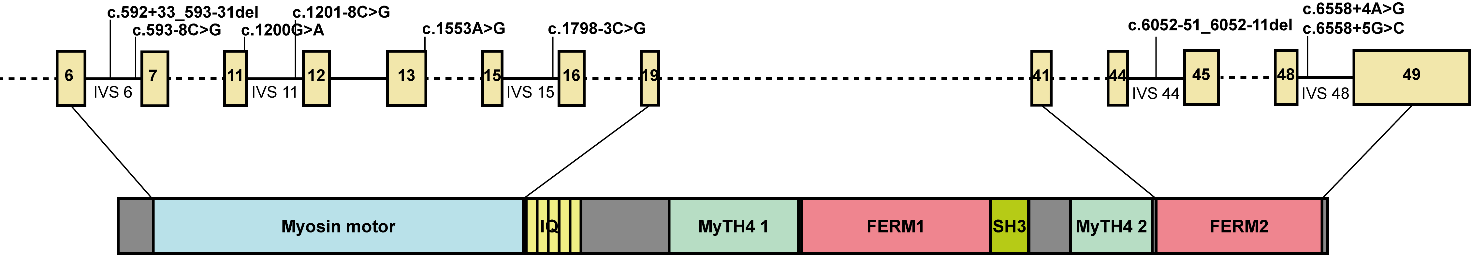
**

**Figure S2. Distribution of variants across *MYO7A* exons and domains.** The variants were mapped on the exon–intron structure of *MYO7A* and its protein domains.


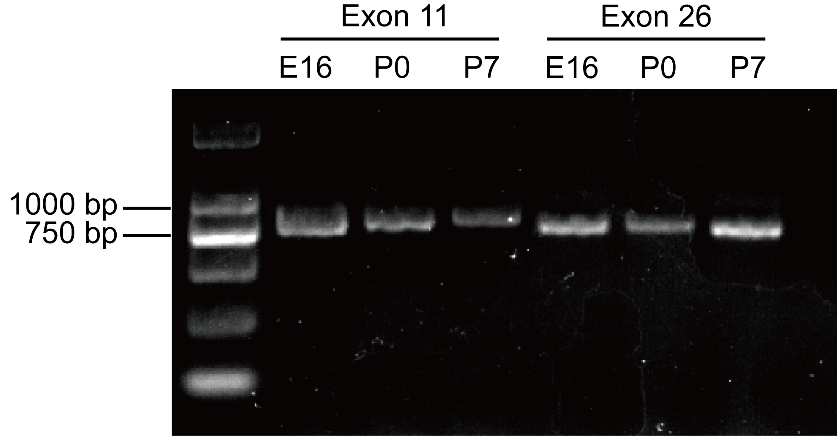


**Figure S3. RT-PCR analysis of cochlear tissue of mice at different time points.** RT-PCR analysis of cochlear tissue from mice at embryonic day 16 (E16), postnatal day 0 (P0), and postnatal day 7 (P7) showing no exon skipping for exon 11 or exon 26. The agarose gel electrophoresis indicates that full-length transcripts are present across all time points, confirming the absence of exon skipping *in vivo*.
